# Hunchback activates Bicoid in Pair1 neurons to regulate synapse number and locomotor circuit function

**DOI:** 10.1016/j.cub.2022.04.025

**Published:** 2022-05-04

**Authors:** Kristen M. Lee, Amanda M. Linskens, Chris Q. Doe

**Affiliations:** Institute of Neuroscience, Howard Hughes Medical Institute, University of Oregon, Eugene, OR 97403, USA

## Abstract

Neural circuit function underlies cognition, sensation, and behavior. Proper circuit assembly depends on the identity of neurons in the circuit (gene expression, morphology, synapse targeting, and biophysical properties). Neuronal identity is established by spatial and temporal patterning mechanisms, but little is known about how these mechanisms drive circuit formation in post-mitotic neurons. Temporal patterning involves the sequential expression of transcription factors (TFs) in neural progenitors to diversify neuronal identity, in part through the initiate expression of homeodomain TF combinations. Here we address the role of the *Drosophila* temporal TF Hunchback and the homeodomain TF Bicoid in the assembly of the Pair1 (SEZ_DN1) descending neuron locomotor circuit, which promotes larval pausing and head-casting. We find that both Hunchback and Bicoid are expressed in larval Pair1 neurons; that Hunchback activates Bicoid in Pair1 (opposite of their embryonic relationship); and that loss of Hunchback function or Bicoid function from Pair1 leads to ectopic presynapse numbers in Pair1 axons and an increase in Pair1-induced pausing behavior. These phenotypes are highly specific, as loss of Bicoid or Hunchback has no effect on Pair1 neurotransmitter identity, dendrite morphology, or axonal morphology. Importantly, loss of Hunchback or Bicoid in Pair1 leads to addition of new circuit partners that may underlie the exaggerated locomotor pausing behavior. These data are the first to show a role for Bicoid outside of embryonic patterning, and the first to demonstrate a cell-autonomous role for Hunchback and Bicoid in interneuron synapse targeting and locomotor behavior.

## eTOC Blurb

Using *Drosophila* larval Pair1 neurons, Lee et al. show that the temporal transcription factor Hunchback activates the homeodomain transcription factor Bicoid. This pathway is required to inhibit ectopic synapses post-mitotically but not for other aspects of Pair1 identity. Hunchback and Bicoid are also required for Pair1 motor circuit function.

## Introduction:

Neural circuit formation underlies the generation of behavior, and aberrant neural circuit development has been associated with many neural disorders, such as autism and ADHD^1^. It is widely accepted that circuit formation requires the assembly of precise interconnectivity between diverse neuron subtypes. Although the mechanisms for generating molecularly and morphologically distinct neurons are well studied (see below), little is known about how these developmental mechanisms regulate ‘higher order’ neuronal properties such as pre- and post-synapse numbers or circuit partner choice.

In *Drosophila*, neuronal identity is specified by the combination of spatial and temporal transcription factors (TFs) acting on neuronal stem cells (neuroblasts, in *Drosophila*). Spatial patterning creates molecularly distinct neuroblasts^2^, followed by each neuroblast sequentially expressing a series of temporal TFs: Hunchback > Kruppel > Pdm (FlyBase: Nubbin/Pdm-2) > Castor, which diversify neurons within each neuroblast lineage^3^. Temporal TFs are known to specify axon and dendrite morphology and targeting^4–8^ as well as behavior^6^. For example, in neuroblast 7-1, the best characterized lineage in the embryo, the zinc-finger temporal TF Hunchback promotes expression of the homeodomain TF Even-skipped which is required for proper motor neuron morphology and connectivity^9–12^; and the combination of Kruppel and Pdm temporal TFs promotes expression of the homeodomain TF Nkx6 (FlyBase: HGTX) which is required for proper ventral projecting motor neuron morphology and connectivity^4,13^. In both cases, transient temporal TF expression activates a homeodomain TF which persists in the post-mitotic neuron to determine neuron morphology and neuromuscular connectivity. Similarly, work from the Hobert lab in C. elegans supports a model in which each of the 302 neurons is specified by a unique combination of homeodomain TFs^14^. Overall, from worms to flies to mammals, temporal TFs activate homeodomain TFs to specify molecular and morphological neuronal identity^15,16–18^.

Although homeodomain TFs are well known to specify these early aspects of motor neuron identity^15,16,17^, their role in specifying later aspects of neuronal identity such as synapse number, position, and connectivity remains poorly understood. To address this question, we utilized the Pair1 (SEZ_DN1) locomotor circuit in *Drosophila*. Pair1 is a GABAergic intemeuron with ipsilateral dendrites and contralateral descending axonal projections^19–21^. The Moonwalker Descending Neurons (MDN) provide inputs to Pair1, and Pair1 sends outputs to A27h neurons in the ventral nerve cord (VNC)^19–22^. When optogenetically activated, the Pair1 neurons induce a pause in forward locomotion and increase in head-casting, in part by inhibiting the A27h neurons, which drive forward locomotion^19–21^. Importantly, we previously reported that the temporal TF Hunchback and the homeodomain TF Bicoid are expressed in Pair1 neurons throughout life^19^, providing candidates to study the transcriptional regulation of Pair1 neuronal identity and connectivity.

Hunchback is the first temporal TF to be expressed in the *Drosophila* embryo and acts transiently to generate early born neurons^3^. In the embryonic CNS, Hunchback is not required to maintain neuronal identity^23^, although it is required to maintain proper dendrite morphology of the mAL interneuron in adult males^24^. Bicoid is a homeodomain TF, yet its expression and function outside the early embryo had not been reported until our recent work^19^. Bicoid is well-known to form an anterior-posterior morphogen gradient that directly activates *hunchback*^25^ to properly pattern the anterior-posterior body axis^26^. Although the role of Hunchback in temporal patterning is conserved in mammals^27^, Bicoid is found only in higher dipteran insects, making it an interesting contributor to insect evolution^28,29^. Here we test the model that the temporal TF Hunchback activates the homeodomain TF Bicoid (opposite of their early embryo relationship), and whether Hunchback and Bicoid play a role in Pair1 neurotransmitter expression, neuron morphology, synapse number, circuit function and behavior. Our data support the emerging model that temporal TFs drive expression of homeodomain TFs that maintain distinct aspects of neuronal identity including synapse number/position, connectivity, and behavior.

## Results

### Hunchback is expressed in Pair1 neurons throughout development.

**Figure 1.**
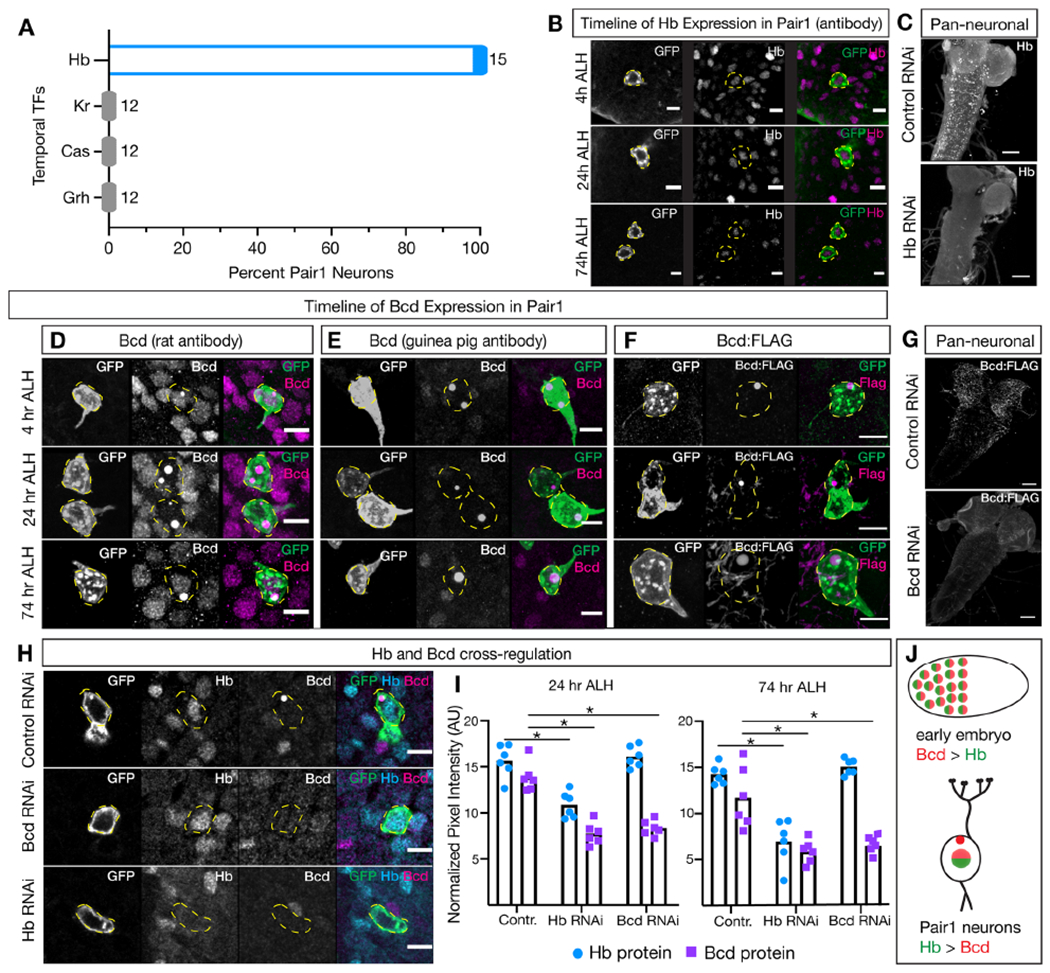
Hunchback activates Bicoid expression in larval Pair1 neurons. (**A**) Percent of Pair1 neurons expressing Hunchback (Hb, left column, blue), Kruppel (Kr, grey, middle left column), Castor (Cas, grey, middle right column) and Grainy head (Grh, grey, right column) via immunostaining. n = 12 −15, reported for each protein. (**B**) Time course of Hunchback (Hb) expression in Pair1 neurons. Pair1 neurons (GFP; left column), Hb antibody (middle column) and merge (right column) at indicated timepoints after larval hatching (ALH). Yellow dashed circles indicate Pair1 cell body. Scale bar, 5μm. Genotype: +;*UAS-myr::GFP*; *R75C02-Gal4*. (**C**) Hb RNAi decreases Hb protein levels. Expression of Hunchback (Hb) in third instar larvae expressing pan-neuronal luciferase RNAi (control) or Hb RNAi. Genotypes: +; *Elav-Gal4/*+; *UAS-Luc RNAi/*+ and +; *Elav-Gal4/*+; *UAS-Hb RNAi/* +. Scale bar, 50μm. (**D-F**) Time course of Bicoid (Bcd) expression in Pair1 neurons at indicated timepoints ALH. (D) Pair1 neurons (GFP, left column) expressing Bcd detected by a rat antibody (middle column). (E) Pair1 neurons (GFP, left column) expressing Bcd detected by a guinea pig antibody (middle column). (F) Pair1 neurons (GFP, left column) expressing a Bcd:FLAG fusion protein (middle column) detected by a FLAG antibody. Genotypes: (D,E) + ;*UAS-myr::GFP*; *R75C02-Gal4; (F)* + ;*UAS-myr::GFP/Bimid-GFP.FPTB*; *R75C02-Gal4*. The Bcd:FLAG also has a GFP tag that was not detectable in our stains. Yellow dashed circles indicate Pair1 cell body. Scale bar, 5μm. (**G**) Expression of Bcd:FLAG in third instar larvae expressing pan-neuronal luciferase RNAi (control) or Bcd RNAi. Genotypes: +; *Bcd-GFP.FPTB/Elav-Gal4*; *UAS-Luc RNAi*/+ and +; *Bcd-GFP.FPTB/Elav-Gal4*; *UAS-Bcd RNAi #1*/+. Scale bar, 50μm. (**H**) Hb RNAi reduces Bcd protein levels but Bcd RNAi does not alter Hb protein levels. Top: control luc RNAi; middle Bcd RNAi; bottom Hb RNAi. Each shown at 24h ALH and stained for the indicated proteins. Yellow dashed circles indicate Pair1 cell body. Scale bar, 5μm. Genotypes: +;*UAS-myr::GFP/* +; *R75C02-Gal4/ UAS-Luc RNAi* and +;*UAS-myr::GFP/* +;*R75C02-Gal4/* *UAS-Bicoid RNAi #2* and +;*UAS-myr::GFP/* +; *R75C02-Gal4/* *UAS-Hb RNAi*. (**I**) Quantification of Hb (blue) and Bed (purple) protein expression within the Pair1 cell body at indicated timepoints ALH. Arbitrary units (AU) normalized to area of the cell body reported. 24 hr ALH Statistics: two-way ANOVA: genotype, F(2, 30) = 48.31, p < 0.001; protein, F(1, 30) = 86.69, p < 0.0001; interaction, F(2, 30) = 15.90, p < 0.0001; Bonferroni’s multiple comparisons between genotypes for each protein: Hb, cont vs Hb RNAi, p < 0.0001, cont vs Bicoid RNAi, p > 0.9999; Bicoid, cont vs Hb RNAi, p < 0.0001, cont vs Bicoid RNAi, p < 0.0001; n = 6 animals. 74 hr AL44 Statistics: two-way ANOVA: genotype, F(2, 30) = 38.49, p < 0.001; protein, F(1, 30) = 42.78, p < 0.0001; interaction, F(2, 30) = 13.73, p < 0.0001; Bonferroni’s multiple comparisons between genotypes for each protein: Hb, cont vs Hb RNAi, p < 0.0001, cont vs Bicoid RNAi, p = 0.9029; Bicoid, cont vs 44b RNAi, p < 0.0001, cont vs Bicoid RNAi, p < 0.0001; n = 6 animals. (**J**) Schematic showing opposite Bcd/Hb genetic interactions in early embryo (top) and neurons (bottom). Hunchback (Hb, green) and Bicoid (Bed, red).

Hunchback is an early temporal TF expressed in the embryonic VNC and the brain^3^. To determine if Pair1 neurons are born during the Hunchback temporal TF window, we labeled Pair1 with GFP using the Pair1-gal4 line^19–21^ and asked whether Pair1 neurons are expressed together with each of the embryonic temporal TFs in newly hatched larvae (0-4 hr ALH). Pair1 neurons expressed Hunchback and none of the other temporal TFs (Figure 1A). We previously showed that Hunchback was expressed in Pair1 neurons in the adult brain^19^, and here we extend these findings to show that Pair1 expresses Hunchback at all larval stages tested (6, 24, and 76 ALH; Figure 1B). Importantly, to demonstrate that the Hunchback RNAi works, pan-neuronal and Pair1-specific expression of the Hunchback RNAi decreased Hunchback levels (Figure 1C, 1H). We conclude that Pair1 is born during the Hunchback temporal window and maintains Hunchback expression into adulthood.

### Bicoid is expressed in Pair1 neurons throughout development.

We previously showed that Bicoid was expressed in Pair1 neurons in the late larval and adult brain^19^, but its expression at multiple larval stages has not been reported. We employed two complimentary strategies to assay for Bicoid expression in Pair1 neurons. For both approaches, we labeled Pair1 neurons with GFP. In the first method, we used two different antibodies against the Bicoid protein and found that Bicoid is expressed in the Pair1 nucleus and cytoplasmic puncta at 6, 24 and 76 hrs ALH (Figure 1D–E). Importantly, both nuclear and puncta antibody staining was abolished by Bicoid RNAi (Figure 1H). In the second method, we imaged an FLAG-tagged endogenously expressed Bicoid protein (Bcd-GFP.FPTB) and found that it is localized to cytoplasmic puncta in Pair1 neurons at 6, 24 and 76 hrs ALH (Figure 1F). Importantly, to demonstrate that the Bicoid RNAi works, pan-neuronal expression of the Bicoid RNAi decreased the expression of the FLAG-tagged Bicoid protein (Figure 1G).

For each Bicoid antibody and the epitope-tagged protein, we observed cytoplasmic puncta, which might be liquid-liquid phase separation^30^ (LPPS; see Discussion). Our results here, taken together with our previous results, show that Bicoid is maintained in the Pair1 neurons from larval development to adulthood, consistent with a model in which the homeodomain Bicoid TF acts to maintain some or all aspects of Pair1 neuronal identity.

### Hunchback activates Bicoid expression in Pair1 neurons.

Bicoid activates Hunchback in the embryonic blastoderm^26^. Here we ask whether Bicoid activates Hunchback in the Pair1 neurons. We labeled Pair1 neurons with GFP and visualized Hunchback and Bicoid expression by antibody staining. In control animals expressing luciferase RNAi in Pair1, both Hunchback and Bicoid show normal expression (Figure 1H “control RNAi”, quantified in 1I). In contrast, when the Bicoid RNAi transgene was expressed in Pair1 neurons, Bicoid expression was significantly reduced in Pair1, but surprisingly Hunchback expression was unchanged (Figure 1H “Bed RNAi”, quantified in 1I). Thus, Bicoid does not activate Hunchback in Pair1.

Next, we tested the converse regulatory relationship: does Hunchback activate Bicoid in Pair1 neurons? We expressed Hunchback RNAi in Pair1 and observed both Hunchback and Bicoid levels were significantly decreased (Figure 1H “Hb RNAi”, quantified in 1I). We saw similar results at 24 and 74 hours after larval hatching (ALH; Figure 1I). Importantly, there are Hunchback binding sites on both the 5’ and 3’ ends of the *bicoid* locus^31,32^, consistent with direct transcriptional expression of *bicoid* by Hb. In addition, we found that pan-neuronal Hunchback knockdown did not appreciably change the number of Bicoid+ neurons, suggesting that Hunchback activation of Bicoid in the Pair1 neurons is highly specific (Figure S1). We conclude that Hunchback activates Bicoid expression in Pair1 neurons, the opposite regulatory relationship as seen in the blastoderm (Figure 1G).

### Hunchback and Bicoid are not required for Pair1 neurotransmitter identity or axon morphology.

**Figure 2.**
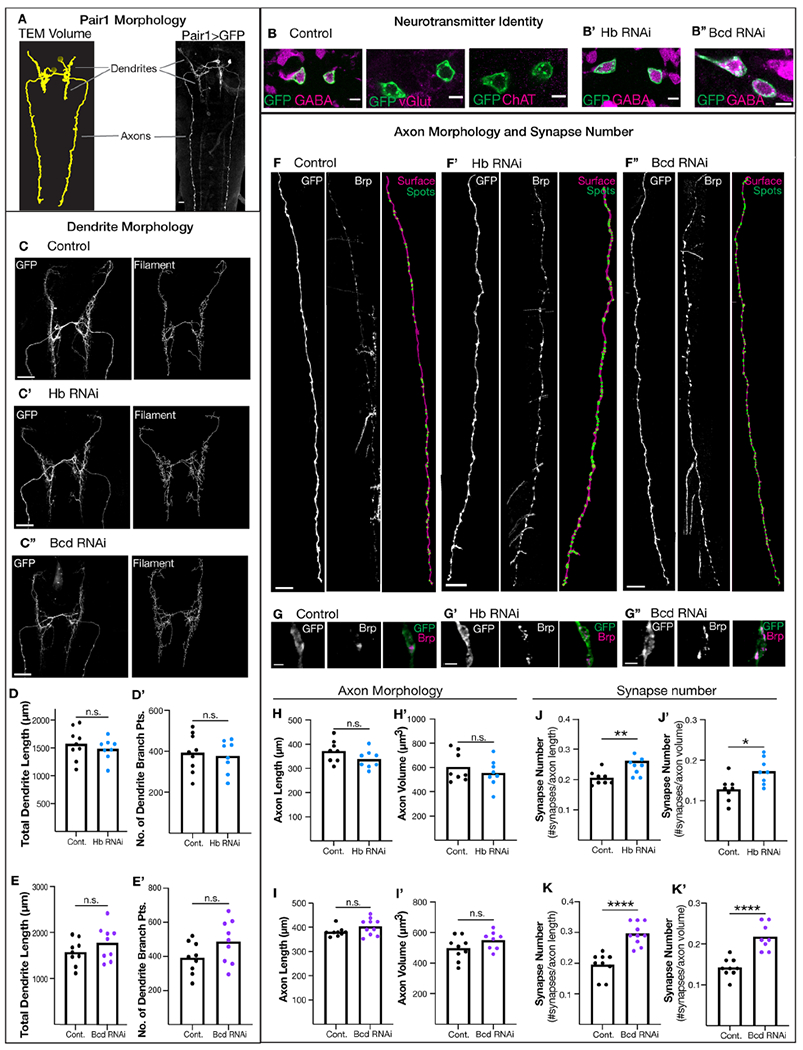
Hunchback and Bicoid regulate synapse number but not neurotransmitter identity, dendrite morphology or axon morphology in Pair1 neurons. (**A**) Morphology of Pair1 neurons. TEM Volume reconstruction (left) compared to GFP expression via Pair1-Gal4 (right). Dendrites and axons labeled. Scale bar, 10 μm. Genotype: +; *UAS-myr::GFP*; *R75C02-Gal4* (**B**) Neurotransmitter expression in Pair1 neurons. Expression of GABA (left column), vGlut (middle column) and ChAT (right column) in Pair1 neurons (B). GABA expression in Hunchback (Hb) knockdown animals (B’) and Bicoid (Bed) knockdown animals (B”) at 74 hr ALH. Scale bar, 5 μm. Genotypes: +; *UAS-myr::GFP*; *R75C02-Gal4/* *UAS-Luc* *RNAi* and +; *UAS-myr::GFP*; R*75C02-Gal4/* *UAS-Hb* RN*Ai* and +; U*AS-myr::GFP;* *R75C02-Gal4/* *UAS-Bicoid* *RNAi* (**C**) Dendrite morphology in animals expressing Luciferase RNAi (Control, C), Hunchback (Hb) RNAi (C’) and Bicoid (Bed) RNAi (C”). Pair1 dendrites (GFP, left column) and reconstructed dendrites (Filaments, right column). Scale bar, 20 μm. (**D**) Total dendrite length in control (black) and Hb RNAi (blue). Statistics: t-test, p = 0.46, n = 8-9 animals. (D’) Number of dendrite branch points in control (black) and Hb RNAi (blue). Statistics: t-test, p = 0.72, n = 8-9 animals. (**E**) Total dendrite length in control (black) and Bed RNAi (purple). Statistics: t-test, p = 0.21,n = 9 animals. (E’) Number of dendrite branch points in control (black) and Bed RNAi (purple). Statistics: t-test, p = 0.09, n = 9 animals. (**F**) Pair1 axons in animals expressing Luciferase RNAi (Control, F), Hunchback (Hb) RNAi (F’) and Bicoid (Bcd) RNAi (F”). Pair1 axons (GFP, left column), pre-synaptic marker Bruchpilot (Brp, middle column) and reconstructed axons (surface, magenta, right column) and reconstructed synapses (spots, green, right column). Scale bar, 15 μm. (**G**) Loss of Bcd or Hb results in ectopic presynaptic puncta at each GFP protrusion (bouton). Pair1 presynaptic puncta at a representative bouton in (G) Luciferase RNAi control, (G′) Hb RNAi, or Bcd RNAi (G”) Scale bar, 2 μm. (**H**) Axon length in control (black) and Hb RNAi (blue). Statistics: t-test, p = 0.1312, n = 8 animals. (H’) Aon volume in control (black) and Hb RNAi (blue). Statistics: t-test, p = 0.4153, n = 8 animals. (**I**) Axon length in control (black) and Bcd RNAi (purple) Statistics: t-test, p = 0.11, n = 9 animals. (I’) Axon volume in control (black) and Bcd RNAi (purple). Statistics: t-test, p = 0.14, n = 8-9 animals. (**J**) Number of synapses normalized to axon length in control (black) and Hb RNAi (blue). Statistics: t-test, p = 0.009, n = 8 animals. (J’) Number of synapses normalized to axon volume in controls (black) and Hb RNAi (blue). Statistics: t-test, p = 0.0117, n = 8 animals. (**K**) Number of synapses normalized to axon length in control (black) and Bcd RNAi (purple). Statistics: t-test, p < 0.0001, n = 9 animals. (K’) Number of synapses normalized to axon volume in control (black) and Bcd RNAi (purple). Statistics: t-test, p < 0.0001, n = 8-9 animals. (C-K) Genotypes: *LexAop-myr::GFP; R75C02-LexA, LexAop-brp-Sh::mCherry/*+*; R75C02-Gal4/UAS-Luc RNAi* and *LexAop-myr::GFP; R75C02-LexA, LexAop-brp-Sh::mCheny/*+*; R75C02-Gal4/UAS-Hb RNAi* and *LexAop-myr::GFP; R75C02-LexA, LexAop-brp-Sh::mCherry/*+*; R75C02-Gal4/UAS-Bcd RNAi*. See also Figure S2

Here we test the role of Hunchback and Bicoid in establishing or maintaining neurotransmitter identity and neuron morphology in Pair1 neurons. Pair1 expresses the neurotransmitter GABA, has ipsilateral dendrites and sends contralateral axons posteriorly down the entirety of the larval VNC (Figure 2A). We knocked down Hunchback and Bicoid individually in Pair1 using validated Hunchback and Bicoid RNAi transgenes (Figure 1) and screened for GABA expression, dendrite morphology and axon morphology at 76 hours ALH. The Pair1-Gal4 driver does not label any additional neurons at 76 hrs, making it an ideal timepoint for this analysis. We first confirmed that Pair1 expresses the neurotransmitter GABA, and not the neurotransmitters glutamate and acetylcholine (Figure 2B). We found that loss of Hunchback and Bicoid in the Pair1 neuron had no detectable effect on GABA levels in Pair1 (Figure 2B). Although unlikely, we cannot rule out that Hunchback and Bicoid knockdown in Pair1 results in expression of a novel neurotransmitter. Next, we assessed dendrite morphology by measuring total dendrite length and the number of dendrite branch points using Imaris image analysis software (Figure 2C, “Filament”). Compared to controls when Hunchback was knocked down dendrite morphology was not changed (Figure 2C’, 2D). Similarly, when Bicoid was knocked down dendrite morphology was also not changed (Figure 2C”, 2E). Lastly, we assessed axon morphology by measuring axon length and volume using Imaris (Figure 2F, “Surface”). Compared to control, when Hunchback was knocked down axon length and volume was not changed (Figure 2F’, 2H). Similarly, when Bicoid was knocked down axon morphology was also not changed (Figure 2F”, 2I). We conclude that reduction in both Hunchback and Bicoid has no effect on Pair1 GABA expression, dendrite morphology or axon morphology.

### Hunchback and Bicoid are required for limiting Pair1 synapse number and position.

Next, we assayed Hunchback and Bicoid RNAi knockdown for changes in synapse number and position. We expressed the well-characterized non-functional presynaptic tag Bruchpilot-Short (Brp)^33,34^ in Pair1 and quantified Brp+ presynaptic puncta along the Pair1 axons, using Imaris to quantify synapse number (Figures 2F, 2J–K). To quantify Pair1 synapse number, we normalized the number of synapses to the axon length and volume. We found that compared to controls, Hunchback knockdown led to a significant increase in presynapse number (Figure 2F′, 2J). Similarly, Bicoid knockdown significantly increased presynapse number compared to control (Figure 2F”, 2K). We also confirmed this result using an independent Bicoid RNAi transgene (Figure S2). Interestingly, the number of presynapses per axonal bouton appears increased when Hunchback and Bicoid was knocked down (Figure 2G), and the ectopic presynapses were preferentially localized to the thoracic region of the Pair1 neuron (Figure 2F; Figure S2). We conclude that Hunchback and Bicoid are required to maintain Pair1 synapse number by preventing the formation of presynapses in the thoracic region of the Pair1 neuron. Note that both Hunchback and Bicoid knockdown are performed constitutively beginning in the embryo, so we can’t make any conclusion about when during development that Hunchback and Bicoid act to regulate synapse number.

### Hunchback and Bicoid act in post-mitotic Pair1 neurons to regulate synapse number.

**Figure 3.**
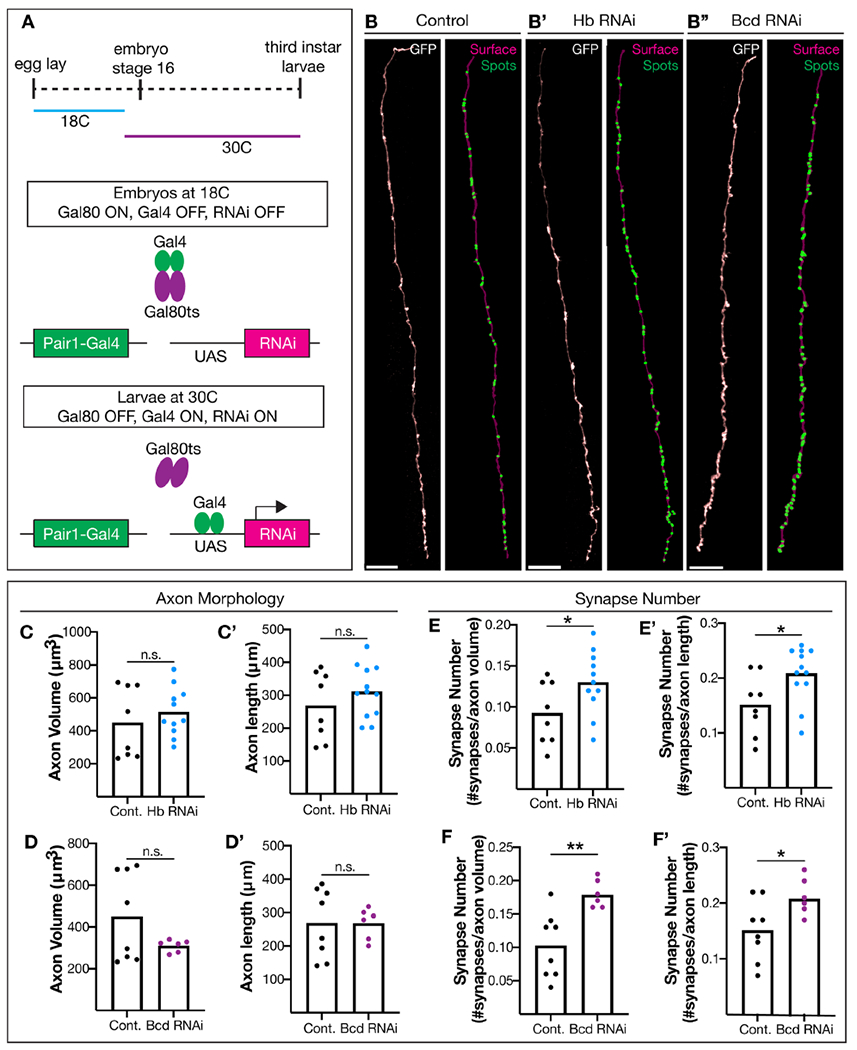
Hunchback and Bicoid function in post-mitotic Pair1 neurons to limit synapse number. (**A**) Schematic of the experimental design; see text for details. (**B**) Pair1 synapse numbers after Luciferase RNAi (control, B), Hb RNAi (B’) or Bcd RNAi (B”). Pair1 axons (GFP, left column), reconstructed axons (surface, magenta) and synapses (spots, green). Scale bar, 20 μm. (**C**) Pair1 axon volume in control (black) and Hb RNAi (blue) animals. Statistics: t-test, p = 0.4432, n = 8-11 animals. (C’) Axon length in control (black) and Hb RNAi (blue) animals. Statistics: t-test, p = 0.3115, n = 8-12 animals. (**D**) Pair1 axon volume in control (black) and Bcd RNAi (purple) animals. Statistics: t-test, p = 0.1393, n = 6-8 animals. (D’) Axon length in control (black) and Bcd RNAi (purple) animals. Statistics: t-test, p = 0.9914, n = 6-8 animals. (**E**) Pair1 synapse numbers normalized to axon volume in control (black) or Hb RNAi (blue) animals. Statistics: t-test, p = 0.0495, n = 8-11 animals. (E’) Number of synapses normalized to axon length in control (black) or Hb RNAi (blue) animals. Statistics: t-test, p = 0.0271, n = 8-12 animals. (**F**) Number of synapses normalized to axon volume in control (black) and Bcd RNAi (purple) animals. Statistics: t-test, p = 0.0037, n = 6-8 animals. (F’) Number of synapses normalized to axon length in control (black) and Bcd RNAi (blue) animals. Statistics: t-test, p = 0.0349, n = 6-8 animals. Genotypes: *LexAop-myr::GFP; R75C02-LexA, LexAop-brp-Sh::mCherry/tub-Gal80ts; R75C02-Gal4/UAS-Luc RNAi* and *LexAop-myr::GLP; R75C02-LexA, LexAop-brp-Sh::mCherry/tub-Gal80ts; R75C02-Gal4/UAS-Hb RNAi* and *LexAop-myr.:GFP; R75C02-LexA, NexAcp-bip-Sh::mCherry|tnb-Gal80ts; R75C02-Gal4/ UAS-Bicoid RNAi*.

To date, Hunchback and Bicoid function have been primarily investigated in the developing embryo^10,26,35^. To investigate a novel role for Hunchback and Bicoid in post-mitotic neurons, we utilized ubiquitously expressed temperature-sensitive Gal80 (tubulin-Gal80ts) to knock down Hunchback and Bicoid at embryo stage 16, when Pair1 neurons are post-mitotic. To accomplish this, we reared animals with the Pair1-Gal4, Gal80ts and Hunchback RNAi transgenes at 18°C; at this temperature the Gal80ts protein is in an active conformation and inhibits Gal4 activation of the RNAi transgene. At embryonic stage 16, the animals were switched to 30°C; at this temperature the Gal80ts protein is in an inactive conformation allowing the Gal4 to drive expression of the RNAi transgene^36^ (Figure 3A). At 76hr ALH we assayed Pair1 axonal volume, length, and synapse density as described previously. When Hunchback and Bicoid were individually knocked down in post-mitotic Pair1 neurons, axon volume and length was not altered compared to control (Figure 3C, 3D), similar to constitutive Hunchback and Bicoid knockdown (see Figure 2). However, Pair1 neuron synapse number was significantly increased when Hunchback and Bicoid were individually knocked down in post-mitotic Pair1 neurons (Figure 3E, 3F). This indicates that Hunchback and Bicoid are required in post-mitotic neurons to limit synapse number. This result is also the first example of the temporal TF Hunchback having an function in post-mitotic axons, and the first example that the homeodomain TF Bicoid has a role beyond the early embryo.

### Hunchback regulates Pair1 connectivity.

**Figure 4.**
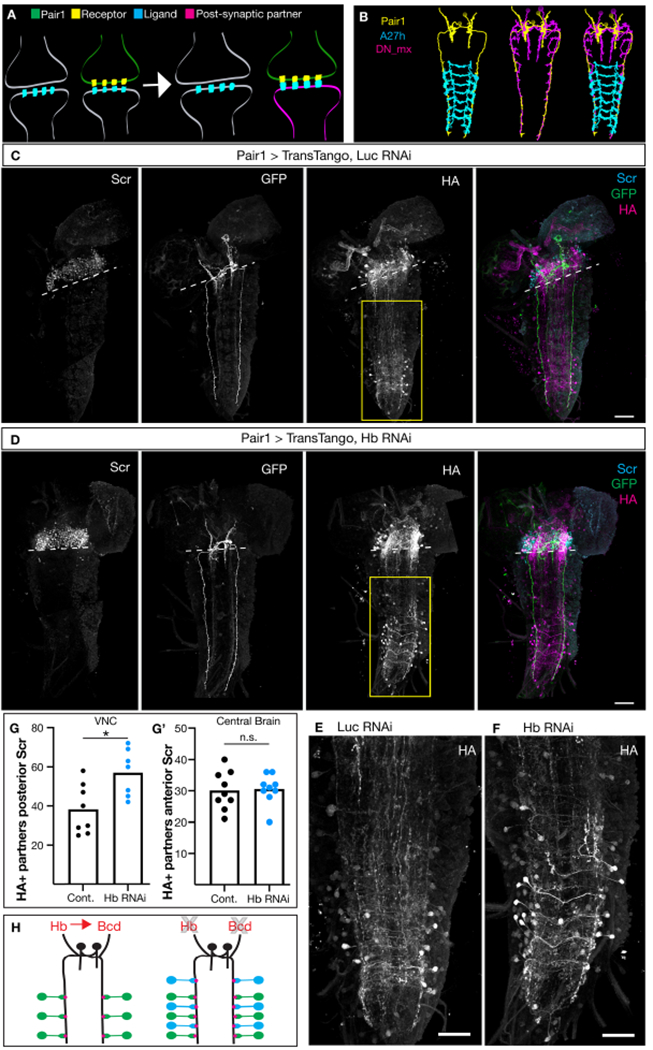
Hunchback is required for proper Pair1 neuron connectivity. (**A**) Schematic of the trans-Tango experimental design; see text for details (**B**) TEM reconstructions of Pair1 (yellow) connectivity with A27h (cyan) and DN_mx (magenta). (**C, D**) trans-Tango was used to visualize Pair1 downstream synaptic partners following Luciferase RNAi (Control, C) or Hb RNAi (D). Sex combs reduced (Scr, left column) defined the central brain and VNC boundary (dashed line), Pair1 neurons (GFP, middle left column), neuronal partners downstream of Pair1 (HA, middle right column) and merge. Compiled Z-projections. Scale bar, 40 μm. (**E, F**) High magnification image of HA+ cell bodies in VNC (yellow box, panel C, D) in control (E) and Hb RNAi (F) animals. Compiled Z-projections. Scale bar, 40 μm. (**G**) Number of HA+ cell bodies posterior to the Scr+ boundary in control (black) and Hb RNAi (blue) animals. Statistics: t-test, p = 0.0015, n = 7-8. (G’) Number of HA+ cell bodies anterior to the Scr+ boundary in control (black) and Hb RNAi (blue) animals. Statistics: t-test, p = 0.86, n = 9. (**H**) Schematic demonstrating Hunchbacks role in mediating both synapse number and synaptic partner selection in Pair1 neurons. Genotypes: *UAS-myr::GFP.QUAS-mtdTomato-3XHA; P(trans-Tango); R75C02-Gal4/ UAS-L/ic RNAi* and *UAS-myr::GFP.QUAS-mtdTomato-3XHA; P(trans-Tango); RJ5C02-Gal4/UAS-Hb RNAi*.

The Pair1 neurons inhibit their direct downstream partners, A27h, to block forward locomotion^20^. Pair1 neurons are also synaptic partners with other neurons in the brain, such as DN_mx neurons^20^. Since Hunchback and Bicoid are required in Pair1 neurons to limit synapse number, we wanted to determine whether the disruptions in circuit function were due to either an increase in synapses within their normal partners, or addition of synapses from novel partners. We used trans-Tango to label direct partners downstream of Pair1 with an HA epitope tag^37^. Specifically, trans-Tango utilizes an artificial signaling pathway that is expressed in all neurons but only activated in downstream neuronal partners of Pair1 (Figure 4A). We expressed the trans-Tango transgene pan-neuronally and Hunchback RNAi transgene in Pair1 neurons, counted the number of HA+ cell bodies labeled in the central brain and VNC, and compared these results to controls with normal Hunchback levels. To quantify synapse number in both the VNC (where Pair1-A27h cell bodies are located, Figure 4B), and the central brain lobes (where Pair1-DN_mx cell bodies are located, Figure 4B), we labeled the subesophageal zone with the Sex combs reduced (Scr) antibody^38^ and counted cell body number above and below the most posterior boundary of the Scr domain (Figure 4C, 4D dashed line). Furthermore, all experiments were conducted at 78 hr ALH when the Pair1 Gal4 driver did not have off-target expression.

Next, we wanted to test the role of Hunchback and Bicoid in establishing or maintaining postsynaptic partner number using trans-Tango. Expressing Bicoid RNAi and trans-Tango led to lethality which precluded assaying synaptic partner numbers. However, combining Hunchback RNAi and trans-Tango allowed development to the late larval stages necessary for this experiment. Interestingly, we found that knocking down Hunchback in Pair1 neurons significantly increased the number of neurons labeled by the trans-Tango transgene in the VNC (Figure 4C–F and quantified in Figure 4G), indicating that specifically in the VNC Pair1 neurons are forming synaptic connections with novel partners. Additionally, knocking down Hunchback had no change on the number of neurons labeled in the central brain (Figure 4G’), indicating that Pair1 is not forming connections with novel neurons in the central brain. These result show that new neurons are connecting to Pair1 in the VNC, although we can’t rule out the possibility that there are also more Pair1-A27h synapses or differences in synaptic strength. We conclude that Hunchback is required for proper Pair1 connectivity in the VNC (Figure 4H).

### Hunchback and Bicoid are required in Pair1 for normal locomotion.

**Figure 5.**
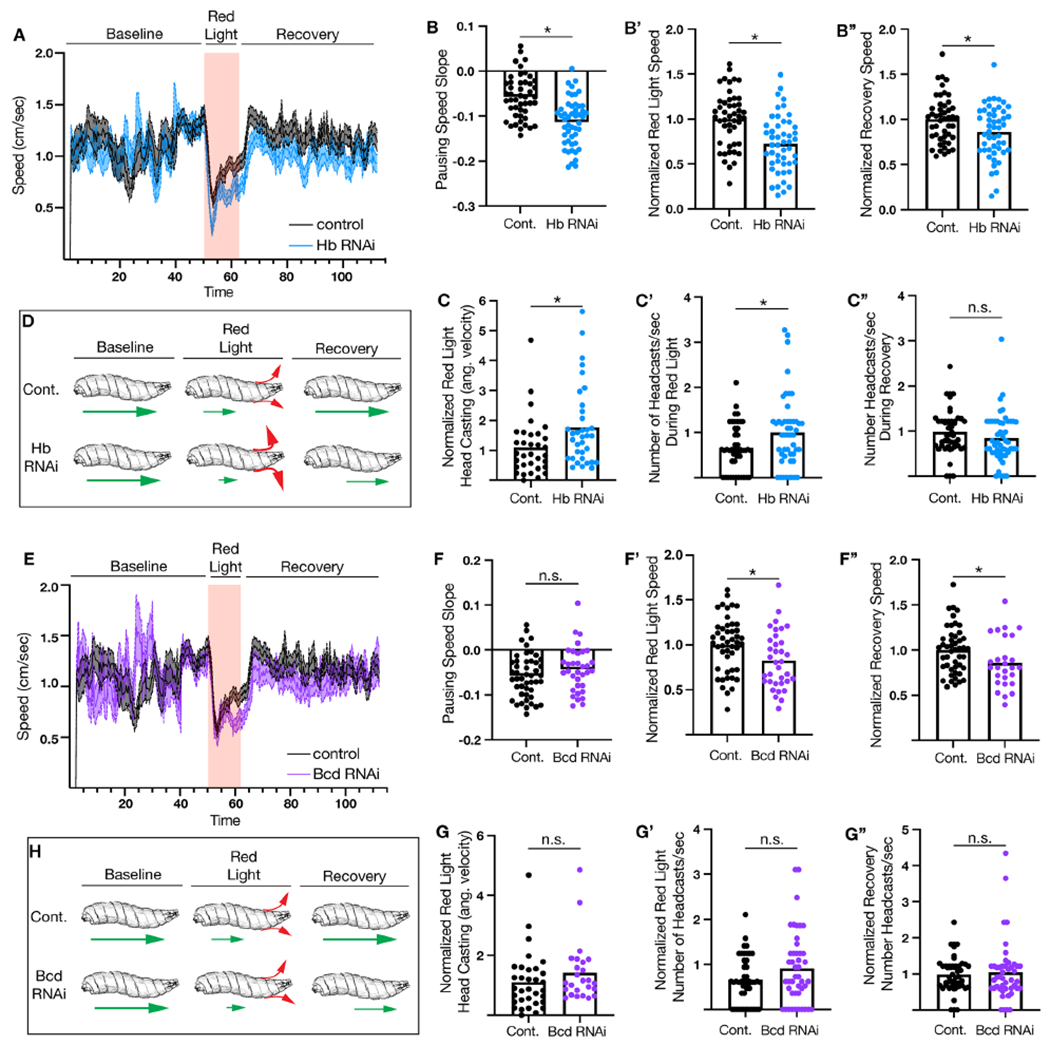
Hunchback and Bicoid are required for normal Pair1-dependent locomotor behavior. (**A**) Speed of animals expressing Luciferase RNAi (Control; black) or Hunchback (Hb) RNAi (blue) larvae over time. Red bar represents red light exposure. (**B**) Pausing speed slope in control (black) or Hb RNAi (blue) animals. Statistics: t-test, p < 0.0001, n = 47-49 animals. (B’) Normalized speed during red light exposure of control (black) and Hb RNAi (blue) animals. Statistics: t-test, p < 0.0001, n = 47-49 animals. (B”) Normalized speed during after the red-light stimulus in control (black) or Hb RNAi (blue) animals. Statistics: t-test, p = 0.0153, n = 47-49 animals. (**C**) Normalized angular velocity of head-casting in control (black) or Hb RNAi (blue) animals. Statistics: t-test, p = 0.02, n = 33-35 animals. (C’) Number of head casts per second during the red-light stimulus in control (black) or Hb RNAi (blue) animals. Statistics: t-test, p = 0.014, n = 47-49 animals. (C”) Number of head casts per second during recovery phase in control (black) or Hb RNAi (blue) animals. Statistics: t-test, p = 0.18, n = 47-49 animals. (**D**) Schematic of control or Hb RNAi animals speed (green arrow) and head casting (red arrow) during the baseline, red-light and recovery phase. (**E**) Speed of animals expressing Luciferase RNAi (Control, black) or Bicoid (Bcd) RNAi (purple) larvae over time. Red bar represents red light exposure. (**F**) Pausing speed slope in control (black) or Bcd RNAi (purple) animals. Statistics: t-test, p = 0.2091, n = 34-47 animals. (F’) Normalized speed during red light exposure of control (black) or Bcd RNAi (purple) animals. Statistics: t-test, p = 0.0145, n = 34-47 animals. (F”) Normalized speed during after the red-light stimulus in control (black) or Bicoid RNAi (purple) animals. Statistics: t-test, p = 0.0304, n = 34-47 animals. (**G**) Normalized angular velocity of head-casting in control (black) or Bcd RNAi (purple) animals. Statistics: t-test, p = 0.23, n = 25-33 animals. (G’) Number of head casts per second during the red-light stimulus in control (black) or Bcd RNAi (purple) animals. Statistics: t-test, p = 0.07, n = 45-47 animals. (G”) Number of head casts per second during recovery phase in control (black) and Bcd RNAi (purple) animals. Statistics: t-test, p = 0.65, n = 46-47 animals. (**H**) Schematic of control or Bcd RNAi animal speed (green arrow) and head casting (red arrow) during the baseline, red-light and recovery phase. Genotypes: *UAS-CsChrimson::mVenus;;RJ5C02-Gal4/UAS-Luc RNAi* and *UAS-CsChrimson::mVenus;;R75C02-Gal4/UAS-Hb RNAi* and *UAS-CsChrimson::mVenus;;RJ5C02-Gal4/UAS-Bicoid RNAi #2*.

It is well-characterized that when the Pair1 neuron is optogenetically activated the larvae pause and perform head casts^19–21^. Here, we knocked down Hunchback and Bicoid individually in Pair1 neurons and assayed locomotion and head-casting before, during, and after activation of Pair1 by the red light gated cation channel CsChrimson^20^. We observed no differences in the speeds of control or Hunchback knockdown larvae before the red-light stimulus was presented (“Baseline” speed, Figure 5A). However, upon Pair1 activation, the Hunchback knockdown larvae showed faster pausing, measured by a significantly steeper negative slope (Figure 5B). We observed that control animals quickly increase their speeds after the initial pause during red light exposure, whereas the Hunchback knockdown animals remained slow. To quantify this, we normalized the speeds of the control and Hunchback knockdown animals during red light exposure to the average control speed. We found that Hunchback knockdown in Pair1 resulted in a significantly slower speed while the red-light stimulus was presented (Figure 5B’). Lastly, we observed that after the red-light exposure, control animals quickly return to a baseline speed (speed before red light stimuli), while the Hunchback knockdown animals took longer. To quantify this, we normalized the speeds of the control and Hunchback knockdown animals after red light exposure to the baseline speed. We found that Hunchback knockdown in Pair1 resulted in an inability to recover speed after red light exposure compared to control (Figure 5B”). We conclude that Hunchback is required in Pair1 for normal Pair1-dependent locomotion (Figure 5D, green arrows represent speed).

Given that reduced speed and head-casting are correlated behaviors in *Drosophila* larvae^21^, we assayed head-casting at timepoints where the larvae speeds were significantly reduced. First, we assayed the angular velocity and number of head-casts during the red-light exposure, when the Hunchback knockdown resulted in significantly slower speed. We found that both the speed of head-casting (Figure 5C) and number of head-casts (Figure 5C’) were significantly increased when Hunchback was knocked down in Pair1 neurons. Next, we assayed the number of head-casts during the recovery phase, when the Hunchback knock down resulted in significantly slower speed. We found no significant difference in the number of head-casts during the recovery phase when Hunchback was knocked down in Pair1 neurons compared to control (Figure 5C”). Taken together, these results suggest that Hunchback functions in Pair1 neurons to regulate pausing and head-casting behavior during Pair1 activation, as well as overall larval speed recovery after pausing (Figure 5D, red arrows represent head-casting).

To determine if the Hunchback knock down phenotype was due to loss of Bicoid, we knocked down Bicoid in Pair1 and assayed locomotion and head-casting (Figure 5E). When Bicoid was knocked down in Pair1, there was no change in pausing speed during Pair1 activation, but there was a significant decrease in overall speed during red light stimulus and the overall speed after the red-light exposure (Figure 5F). Additionally, when Bicoid was knocked down in Pair1, there was no change in the angular velocity or number of head-casts during the red-light stimulus or the recovery phase (Figure 5G). These results suggest that Bicoid expression in Pair1 is only required for overall speed during Pair1 activation and speed recovery after Pair1 activation (Figure 5H). Taken together, knock down of Hunchback or Bicoid have similar effects on larval locomotor behaviors, with Hunchback knock down having a stronger phenotype. This could be due to Hunchback regulating additional genes beyond Bicoid (see Discussion). In conclusion, our data suggest that Hunchback and Bicoid are required in Pair1 neurons for normal Pair1 circuit function.

## Discussion

Our results show that Hunchback activates Bicoid in post-mitotic Pair1 neurons, where it regulates specific and important aspects of neuronal identity - synapse number, synapse density, and connectivity. When Hunchback or Bicoid levels are decreased, synapse density is increased, with a corresponding disruption of the function of the Pair1 locomotor neural circuit. This work demonstrates a novel role for Hunchback and Bicoid – functioning post-mitotically to regulate synapse number and to ensure proper circuit function. Importantly, this work also reproduces a phenotype previously seen in *C. elegans* – a single homeobox gene (*unc-4*) specifically regulates synaptic connectivity but not other aspects of neuronal identity^18^. Interestingly, *unc-4* expression is also regulated by a non-homeodomain transcription factor^39,40^, suggesting that this regulatory pathway may be conserved between species to specify highly-specific aspects of neuronal identity.

Unlike most early-born neurons in the VNC that only transiently express Hunchback^23^, and Bicoid which is only expressed in the first few hours of embryogenesis, the Pair1 neuron maintains both Hunchback and Bicoid expression into the adult. This suggests that a Pair1-specific regulatory mechanism may be leading to the persistent Hunchback and Bicoid expression and function. Given that the Pair1 neuron persists into adulthood, still expresses Hunchback and functions within a similar locomotor neural circuit^19^, we hypothesize that Hunchback and Bicoid expression may be required in Pair1 neurons throughout life for the maintenance of the Pair1 locomotor neural circuit.

To our surprise, Bicoid protein expression in larval Pair1 neurons was often detected in one or more spherical puncta located in the cytoplasm; this was observed with two independent Bicoid antibodies and a third FLAG-tagged Bicoid protein and was abolished by Bicoid RNAi. Given that Bicoid contains highly disordered regions with an abundance of glutamine and glycine, the spherical puncta may represent a phase-separation condensate^30^, perhaps to keep nuclear Bicoid levels low. Interesting, Bicoid does not form spherical puncta outside of the larvae^19^. Further investigation is needed to understand nature of the Bicoid cytoplasmic puncta, but these studies have the potential to elucidate a novel role for phase-separation in mature neurons.

Previous work showed that Bicoid activates *hunchback* in the early embryo^26^. Our study is the first we are aware of to demonstrate the reverse: that Hunchback can promotes Bicoid expression *in vivo*. Hunchback may regulate Bicoid directly or indirectly; supporting the former possibility are the findings that Hunchback protein binds two distinct regions at the 3’ and 5’ end of the *bicoid* locus^31,32^. Alternatively, Hunchback may act indirectly by promoting Bicoid phase separation in larval neurons. Regardless, this finding supports our initial hypothesis that temporal transcription factors, like Hunchback, can activate homeodomain transcription factors, like Bicoid, to specify some or all aspects of neuronal identity. Other morphogens have been previously associated with establishing properties of neuronal identity ^41^, further suggesting that early developmental transcription factors may be important regulators of neuronal identity, connectivity and circuit function in general.

We found that Hunchback and Bicoid had no detectable role in regulating dendrite morphology, axon morphology nor GABA expression, key aspects of Pair1 neuronal identity. Yet we found both Hunchback and Bicoid are required for maintaining synapse number and functional connectivity of the Pair1 neuron. Our trans-Tango experiments show that reduced Hunchback levels resulted in the addition of new synaptic partners of Pair1, although we can’t exclude the possibility that these may be normal partners that are too weak to see in controls. Although we did not formally identify the novel neuronal partners, we utilized the *Drosophila* larvae TEM volume to speculate that Pair1 could be synapsing with the A27h neurons located in the thoracic region^42^. Given that A27h neurons are involved in forward locomotion^20^, additional thoracic A27h neurons synapsing onto, and therefore being inhibited by Pair1 activation, could explain the increased pausing phenotype observed when Hunchback in knocked down in Pair1. Alternatively, abdominal A27h neurons could be forming more synapses with Pair1 in the posterior axonal regions.

Interestingly, it appears that Bicoid is not the only homeodomain TF functioning downstream of Hunchback in Pair1. When Hunchback is knocked down in Pair1, pausing speed is increased, head-casting is increased and recovery speeds are decreased. However, Bicoid knockdown only replicated the decreased recovery speed phenotype (Figure 5); this suggests that another homeodomain TF may be functioning downstream of Hunchback to regulate pausing speed and head-casting. The data presented here begin to support this hypothesis, but additional work is needed to identify other homeodomain TFs functioning downstream of Hunchback.

Our work is the first, to our knowledge, to demonstrate a role for Hunchback and Bicoid in post-mitotic neurons to regulate synapse number, connectivity, and circuit function. Our results raise the question of which is the more ancestral function of these two TFs: in segmentation, temporal patterning in neuroblasts, or post-mitotic neuronal circuit maintenance?

## Supplementary Material

2

## Figures and Tables

**Table:** KEY RESOURCE TABLE

REAGENT OR RESOURCE	SOURCE	IDENTIFIER
***Antibodies***		
Chicken polyclonal anti-GFP	Abcam, Eugene, OR	RRID: AB_13970
Rabbit polyclonal anti-mCherry	Novus, Centennial, CO	Cat # NBP2-25157
Rat monoclonal anti-HA (3F10)	Sigma, St. Louis, MO	SKU: 11867423001
Rat monoclonal anti-Flag	Novus, Centennial, CO	Cat # NBP1-06712
Mouse monoclonal anti-Scr	DSHB, Iowa City, IA	RRID: AB_528462
Rabbit polyclonal anti-GABA	Sigma, St. Louis, MO	Cat # A2052
Rat polyclonal anti-Bcd	John Reinitz, University of Chicago, IL	
Guinea Pig anti-Bcd	John Reinitz, University of Chicago, IL	
Rabbit anti-Hb	Made in lab previously^41^	
Secondary Antibodies	Jackson ImmunoReasearch, West Grove, PA	
***Chemicals, peptides, and recombinant proteins***		
All-trans-retinal	Sigma-Aldrich	R2500-100MG
***Experimental models: Organisms/strains***		
UAS-CsChrimson::mVenus	Vivek Jayaraman, Janelia Research Campus	
LexAop-brp-Sh::mCherry	Gift from Takashi Suzuki, Toyko Institute of Technology	
R75C02-Gal4 (referred to as Pair1-Gal4)	Bloomington Drosophila Stock Center	BDSC 39886
R75C02-LexA	Bloomington Drosophila Stock Center	BDSC 54365
UAS-myr::GFP	Bloomington Drosophila Stock Center	BDSC 32198
LexAop-myr::GFP	Bloomington Drosophila Stock Center	BDSC 32211
UAS-Luc RNAi	Bloomington Drosophila Stock Center	BDSC 31603
UAS-Hunchback RNAi	Bloomington Drosophila Stock Center	BDSC 34704
UAS-Bicoid RNAi	Bloomington Drosophila Stock Center	BDSC 33886
UAS-Bicoid RNAi #2	Bloomington Drosophila Stock Center	BDSC 35478
Bicoid-GFP.FPTB	Bloomington Drosophila Stock Center	BDSC 67654
Tubulin-Gal80ts	Bloomington Drosophila Stock Center	BDSC 7019
UAS-myrGFP.QUAS-mtdTomato-3XHA; trans-Tango (referred to TransTango)	Bloomington Drosophila Stock Center	BDSC 77124
***Software and algorithms***		
FIMtrack	WWU Munster	http://www.uni-muenster.de/Informatik.AGRisse/media/fim-media.html
FIJI	ImageJ	http://imagej.net/software/fiji/
Prism 9	GraphPad	https://www.graphpad.com/
Imaris 9.5	Oxford Instruments	https://imaris.oxinst.com

## Highlights:

- Hunchback activates Bicoid in *Drosophila* larval Pair1 neurons
- Hunchback and Bicoid inhibit ectopic synapse formation post-mitotically
- Hunchback and Bicoid do not regulate other aspects of Pair1 neuronal identity
- Hunchback and Bicoid promote larval pausing and head-casting
