# Supplementary material for: Hunchback activates Bicoid in Pair1 neurons to regulate synapse number and locomotor circuit function
Source: Curr Biol. Author manuscript; Available in PMC 2023 Jun 6. (PMC9178783; doi:10.1016/j.cub.2022.04.025)
Supplement: 2 [file NIHMS1805439-supplement-2.pdf]

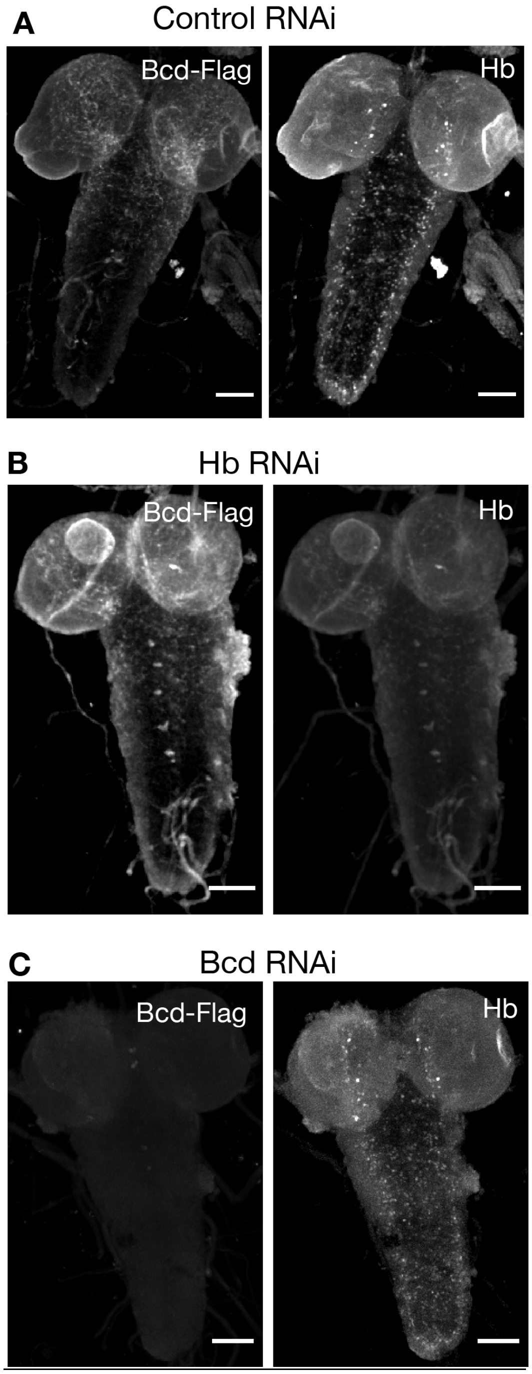

Figure S1. Pan-neuronal expression of Hunchback and Bicoid. Related to Figure 1.
(A) Expression of Hunchback antibody and Bcd-Flag in third instar larvae expressing pan-neuronal luciferase RNAi (control RNAi).
(B) Hunchback RNAi decreases pan-neuronal Hunchback levels but not Bicoid levels. Expression of Hunchback antibody and Bcd-Flag in third instar larvae expressing pan-neuronal Hunchback RNAi (Hb RNAi).

(C) Bicoid RNAi decreases pan-neuronal Bicoid levels but not Hunchback levels. Expression of Hunchback antibody and Bcd-Flag in third instar larvae expressing pan-neuronal Bicoid RNAi (Bcd RNAi).
Genotypes: +*; Bcd-GFP.FPTB/Elav-Gal4; UAS-Luc RNAi*/+ and +*; Bcd-GFP.FPTB/Elav-Gal4; UAS-Hb RNAi*/+ and +*; Bcd-GFP.FPTB/Elav-Gal4; UAS-Bcd RNAi #1*/+. Scale bar, 50µm.

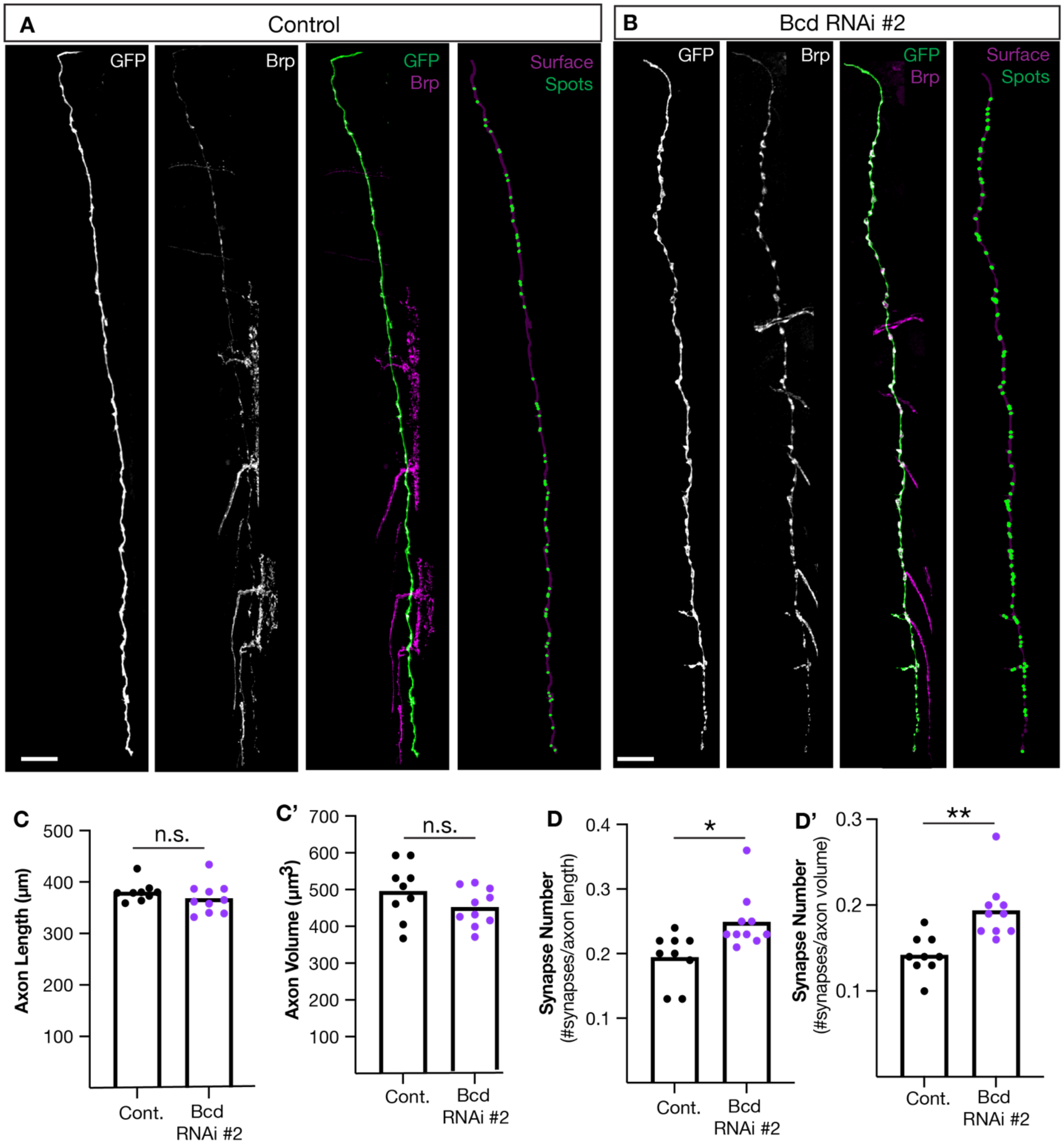

Figure S2. Bicoid regulates synapse number, but not axon morphology, in Pair1 neurons, Related to Figure 2.

(**A-B**) Pair1 axons in animals expressing Luciferase RNAi (Control, A) and Bcd RNAi #2 (B; distinct from Bcd RNAi in Figure 2). Pair1 axons (GFP, left column), presynaptic marker Bruchpilot (Brp, left middle column), merge (right middle column) and reconstructed axon and presynapses (surface and spots, right column). Scale bar, 20 µm

(**C**) Axon length in control (black) or Bcd RNAi #2 (purple) animals. Statistics: t-test, p = 0.31, n = 9-10 animals. (C') Axon volume in control (black) or Bcd RNAi #2 (purple) animals. Statistics: t-test, p = 0.16, n = 9-10 animals.

(**D**) Number of synapses normalized to axon length in control (black) or Bcd RNAi #2 (purple) animals. Statistics: t-test, p = 0.01, n = 9-10 animals (D') Number of synapses normalized to axon volume in control (black) of Bcd RNAi #2 (purple) animals. Statistics: t-test, p = 0.001, n = 9-10 animals. Genotypes: *LexAop-myr::GFP; R75C02-LexA, LexAop-brp-Sh::mCherry/+; R75C02-Gal4/ UAS-Luc RNAi* and *LexAop-myr::GFP; R75C02-LexA, LexAop-brp-Sh::mCherry/+; R75C02-Gal4/ UAS-Bicoid RNAi #2*.
